# An 82-year-old Caucasian man with a ductal prostate adenocarcinoma with unusual cystoscopic appearance: a case report

**DOI:** 10.1186/1752-1947-5-4

**Published:** 2011-01-11

**Authors:** Stavros Sfoungaristos, Ioannis S Katafigiotis, Stavros I Tyritzis, Adamantios Kavouras, Panagiotis Kanatas, Anastasios Petas

**Affiliations:** 1 Department of Urology, University Hospital of Patras, (Rio), Patra (26504), Greece; 2 Department of Urology, Athens University Medical School-LAIKO Hospital, (Agiou Thoma), Athens (11527), Greece; 3 Department of Urology, General Hospital of Korinthos (Leoforos Athinon), Korinthos (20100), Greece; 4 Department of Urology, General Hospital of Rhodes (Agioi Apostoloi), Rhodes (85100), Greece

## Abstract

**Introduction:**

Ductal adenocarcinoma is a rare variety of the common acinar adenocarcinoma. It usually presents with refractory symptoms, and during cystoscopy, it is seen as an exophytic lesion at the area of the verumontanum.

**Case presentation:**

An 82-year-old Caucasian man was diagnosed with ductal adenocarcinoma of the prostate after undergoing transurethral resection of the prostate for urinary retention. Immunohistochemistry confirmed the nature of the tumor. The patient was treated with triptorelin, 3.75 mg once/month, and bicalutamide, 50 mg 1 × 1. The serum prostate-specific antigen at three, six and 12 months after transurethral resection of the prostate was 0.1 ng/ml. The patient remains asymptomatic, and he entered a six-month follow-up protocol.

**Conclusion:**

Ductal adenocarcinoma often involves the central ducts of the gland and may present as an exophytic papillary lesion in the prostatic urethra. This is why it usually presents with refractory symptoms. The outcome for men with prostatic ductal adenocarcinoma is, in most studies, worse than the outcome for men with prostatic acinar adenocarcinoma. Aggressive management is indicated, even with low-volume metastatic disease.

## Introduction

Ductal carcinoma of the prostate was originally identified by Melicow and Pachter in 1967. Thought initially to be a neoplastic proliferation of remnant paramesonephric tissue, it was given the name endometrioid carcinoma. More extensive pathologic analysis, including ultrastructural studies, determined that these tumors, however, originate from the prostate and are now more correctly termed ductal carcinoma, as a variant of the common acinar adenocarcinoma. We present a case of ductal adenocarcinoma, which, during cystoscopy, was missing the characteristic exophytic lesion and looked like a flat, reddish, edematous area at the prostatic urethra.

### Case presentation

An 82-year-old Caucasian man arrived at the emergency department of our hospital complaining of painless, total, macroscopic hematuria starting 24 hours ago. His medical history includes some lower urinary tract symptoms, starting six years ago, insulin-dependent diabetes mellitus, and an episode of stroke five years ago. Clinical examinations were normal, and digital rectal examination (DRE) was negative for pathologic findings. The estimated prostate volume was 70 ml. The laboratory findings were normal, and total serum PSA was 3.7 ng/ml.

At the abdominal ultrasound, the prostate volume was calculated as 65 ml, and the residual volume was 45 ml. During cystoscopy, the bladder mucosa had a normal macroscopic appearance and an enlarged prostatic middle lobe with small areas of hemorrhage was noted.

The patient left the hospital with finasteride, 5 mg 1 × 1, and tamsulosin, 0.4 mg 1 × 1. Three months later, the serum PSA was 2.9 ng/ml.

**Figure 1:**
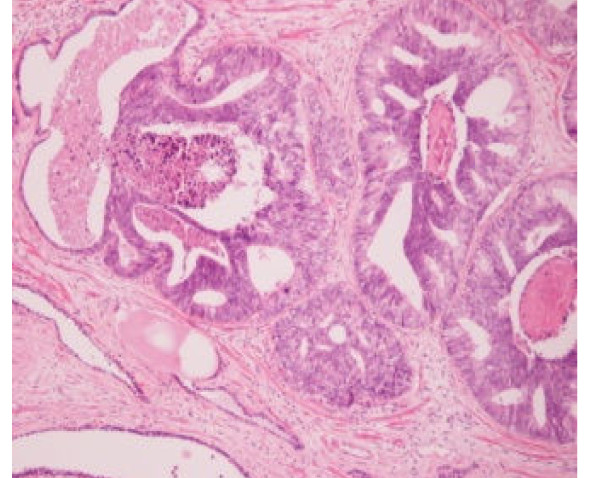
**Ductal adenocarcinoma of the prostate**.

**Figure 2:**
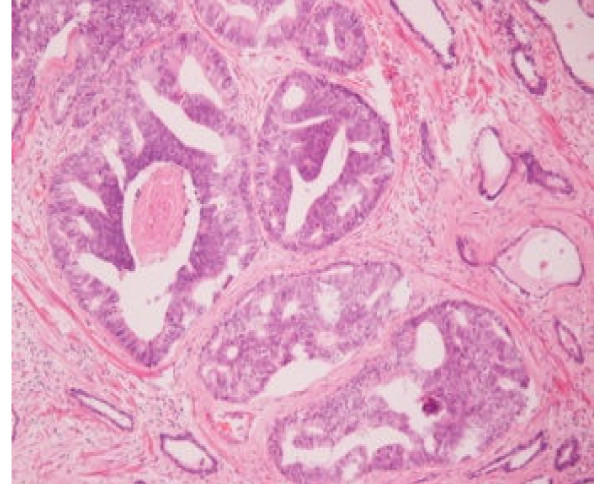
**Ductal adenocarcinoma of the prostate**.

Five months later, the patient returned to the emergency department for urinary retention. An 18F Foley catheter was inserted, and 15 days later, the patient had a transurethral resection of the prostate (TURP). During the operation, we found a diffuse redness of the whole prostate, especially at the area of the prostatic urethra proximal to the verumontanum. The redness involved the bladder neck, the area of the triangle, and the left lateral bladder wall. The same area was characterized by diffuse edema. The prostatic lateral and middle lobes were removed and cold-cup biopsies were taken from the edematous area of the bladder neck and lateral wall. The histologic examination showed ductal prostatic adenocarcinoma (Figures 1 and 2). The CT scan of upper and lower abdomen and thorax and the bone scan were negative for metastasis.

The patient was treated with triptorelin, 3.75 mg once/month and bicalutamide, 50 mg 1 × 1. The serum PSA at three, six, and 12 months after TURP was 0.1 ng/ml. The patient remains asymptomatic, and he entered a six-month follow-up protocol.

## Discussion

This tumor accounts for fewer than 1% of prostatic adenocarcinomas (as a dominant pattern) and has been referred to under a number of different names including endometrioid and papillary carcinoma [1]. The incidence of ductal adenocarcinoma, including both pure ductal and mixed ductal-acinar adenocarcinomas, is 3.2% of all prostatic carcinomas. Clinically, ductal adenocarcinoma often involves the central ducts of the gland and may present as an exophytic papillary lesion in the prostatic urethra. For this reason, they are often seen in transurethral resection (TUR) specimens and at radical prostatectomy (RP), and are less often found in needle biopsies. When diagnosed by needle biopsy, more than 50% of the patients will have high-volume disease with a higher frequency of advanced pathologic stage and a shorter time to progression. The tumor presents in elderly men (age range, 65 to 87 years) with hematuria or obstructive symptoms due to a prostatic urethral mass [2]. The digital rectal examination is usually abnormal and often suggestive of malignancy, with an enlarged and nodular prostate gland. PSA is expressed by ductal carcinoma cells but is not elevated in all patients. The possibility of PSA production in an associated acinar component also makes interpretation of the PSA difficult and, as such, a normal serum PSA before surgery does not allow prediction of the final pathologic stage. PSA cannot reliably be used to risk stratify patients [3]. A recent report suggests that because most ductal adenocarcinomas secrete PSA, they may be more likely to produce unusual serum markers, such as carcinoembryonic antigen [4]. Ductal adenocarcinomas have a more aggressive clinical course and must be diagnostically separated from pure acinar adenocarcinoma. Varying reports concerned serum PSA measurements in cases with a predominant ductal pattern, with some indicating a lower level than might otherwise be expected.

The clinical macroscopic appearance of ductal adenocarcinoma by cystourethroscopy, is, in many cases, that of an exophytic, villous/polypoid growth, with white fronds of "worm-like" tumor protruding into the urethra at or near the verumontanum. The prostatic urethra can also appear narrowed, nodular, or normal. Ductal adenocarcinoma spreads outside the prostate gland in the same fashion as pure acinar adenocarcinoma. The papillary and/or cribriform growths can involve periprostatic soft tissue, seminal vesicles, pelvic lymph nodes, and distant sites, including lung and bone. Ductal adenocarcinoma appears to have a propensity to metastasize to testis, penis, and lung [4].

The outcome for men with prostatic ductal adenocarcinoma is, in most studies, worse than the outcome for men with prostatic acinar adenocarcinoma. Survival and response to therapy appear to be related to stage. Many patients with prostatic ductal adenocarcinoma present with large tumors and advanced stage, including bony metastasis; this may account for the relatively poor prognosis. Some patients respond to radical prostatectomy, hormonal therapy, and radiotherapy. Factors other than stage that predict outcome have not been well-characterized. Aggressive management is indicated, even with low-volume metastatic disease.

## Conclusion

Ductal adenocarcinoma accounts for less than 1% of prostatic adenocarcinomas as a dominant pattern. Ductal adenocarcinomas have a more-aggressive clinical course and must be diagnostically separated from pure acinar adenocarcinoma. Ductal adenocarcinoma often involves the central ducts of the gland and, for this reason, they are often seen in transurethral resection (TUR) specimens. It usually presents with refractory symptoms, and during cystoscopy, it is seen as an exophytic lesion at the area of the verumontanum. In our case, the cystoscopic appearance was unusual, and during the operation, we found a diffuse redness at the whole prostate and especially at the area of the prostatic urethra proximal to the verumontanum.

Aggressive management is indicated, even with low-volume metastatic disease.

## Competing interests

The authors declare that they have no competing interests.

## Consent

Written informed consent was obtained from the patient for publication of this case report and accompanying images. A copy of the written consent is available for review by the Editor-in-Chief of this journal.

## Authors' contributions

SS gathered patient data and drafted the manuscript. ISK drafted and revised the manuscript and gathered reference articles. SIT drafted and revised the manuscript. AK gathered patient data and drafted the manuscript. PK gathered patient data. AP performed the surgical operation and supervised the manuscript. All authors read and approved the final manuscript.
